# Advancing Neurological Health: Insights into Aging, Immunity, and Vascular Dynamics

**DOI:** 10.14336/AD.2024.0423

**Published:** 2024-05-07

**Authors:** Tongtong Xu, Jing Ye, Lin Gan, Shiyu Deng, Yiyan Guo, Yaohui Tang

**Affiliations:** Shanghai Jiao Tong Affiliated Sixth People’s Hospital, School of Biomedical Engineering, Shanghai Jiao Tong University, Shanghai 200030, China

**Keywords:** aging, blood flow, immunity, microglia, neurological disorders

## Abstract

This editorial provides an overview of recent advancements in the understanding and treatment of neurological disorders, focusing on aging, immunity, and blood flow, as featured in this special issue. The first section explores the importance of identifying biomarkers of aging and aging-related diseases, such as Alzheimer's Disease, highlighting the emerging role of saliva-based biomarkers and the gut-brain axis in disease diagnosis and management. In the subsequent section, the dysregulated immune systems associated with aging are discussed, emphasizing the intricate landscape of the immune system during aging and its bidirectional relationship with neuroinflammation. Additionally, insights into the involvement of Myeloid-Derived Suppressor Cells (MDSCs) in Multiple Sclerosis (MS) pathogenesis are presented. The third section examines the role of microglia in neuroinflammation and various neurological diseases, including age-related macular degeneration (AMD) and Tuberculous Meningitis (TBM). Furthermore, the therapeutic potential of stem cell and extracellular vesicle-based therapies for stroke is explored, along with molecular mechanism of how inflammation regulates cerebral and myocardial ischemia. Finally, the importance of blood flow in maintaining vascular health and its impact on neurological disorders are discussed, highlighting the potential of novel assessment methods for optimizing patient care. Overall, this special issue offers valuable insights into the complex mechanisms underlying neurological disorders and identifies potential avenues for therapeutic intervention.

## Biomarker of Aging and age-related diseases

Identifying biomarkers of aging and age-related diseases including Alzheimer's Disease is crucial for achieving early disease diagnosis. Through a comprehensive review, Tao and colleagues delve into known molecular, cellular, and physiological biomarkers of aging, shedding light on the complex mechanisms underlying the aging process, highlighting the applications of the currently available technologies in aging research [1]. For age related diseases such as Alzheimer's Disease (AD), Antequera et al underscored the emerging role of saliva-based biomarkers in AD diagnosis, offering advantages such as non-invasiveness and potential for early screening. Their findings contribute to identifying salivary lactoferrin as a reliable biomarker for early detection and management of Alzheimer's disease. However, further validation and larger-scale studies are warranted [2]. Recently, the importance of the gut-brain axis has been well recognized in AD, but the specific mechanisms by which the gut microbiota communicates with the brain and influences cognition and function remain unclear. Qu et al summarized multiple pathways involved in the gut microbiome's impact on AD and proposed treatment strategies based on targeting the gut microbiome. By addressing these challenges and proposing innovative treatment strategies, the review provides valuable insights into the complex interplay between the gut microbiota and AD pathogenesis, highlighting the potential for targeting the gut microbiome as a promising therapeutic avenue for treating AD [3].

Deinhardt-Emmer and colleagues investigated the role of dipeptidyl peptidase 4 (DPP4) in cellular senescence and its potential association with severe coronavirus disease 2019 (COVID-19) and respiratory infections. DPP4, also known as CD26, is a serine exopeptidase that cleaves X-proline or X-arginine amino acids from the N-terminal side, resulting in the loss of N-terminal dipeptides with pro-inflammatory effects. The researchers observed an association between severe COVID-19 and DPP4, proposing DPP4 plays a major role as part of the senescence-associated secretory phenotype (SASP) during cellular senescence, impacting respiratory infections and potentially contributing to the severity of COVID-19 in older individuals [4].

## Aging and Immunity

The immune system plays a crucial role in maintaining tissue homeostasis and the normal physiological functions of the body. Aging and age-related diseases result in dysregulated immune system, characterized by a chronic inflammatory state and impaired cell-mediated functions. The study by Jun et al. sheds light on the intricate landscape of the immune system during aging, particularly focusing on the phenomenon of multiple T cell receptor (TCR) expression using single-cell RNA sequencing. Their findings reveal a higher prevalence of T cells expressing multiple β chains compared to those expressing multiple α chains in older individuals. This finding underscores the complexity of T cell receptor diversity and raises questions about the mechanisms governing T cell development and selection during aging [5]. Chen and colleagues highlighted the bidirectional relationship between neuroinflammation and the immune system, emphasizing how immunosenescence and inflammation can influence each other, and discusses how the immune system and its cellular components and molecular mechanisms are affected by age following a stroke. The study contributes to a deeper understanding of the mechanisms underlying aging-related neurological conditions and identifies potential avenues for intervention and prevention [6].

Myeloid-derived suppressor cells (MDSCs) are a heterogeneous population of immature myeloid cells that play a critical role in regulating immune responses, which were first described more than 20 years ago in patients with cancer. They are generated in various pathological conditions, such as cancer, inflammation, and infection. MDSCs exert immunosuppressive functions by inhibiting the activation and proliferation of T cells, natural killer cells, and other immune cells. Accumulating evidence suggests that MDSCs accumulate in inflamed tissues and lymphoid organs of both multiple sclerosis (MS) patients and Experimental autoimmune encephalomyelitis (EAE) mice, suggesting a complex interplay between MDSCs and the immune response in MS/EAE pathogenesis. Jiang et al summarized our current understanding of MDSC subsets and their potential roles in MS/EAE pathogenesis. Additionally, it discusses the potential utility of MDSCs as biomarkers and cell-based therapies for MS, highlighting associated obstacles and challenges. This review contributes to our understanding of the involvement of MDSCs in MS/EAE and provides insights into their potential as therapeutic targets and biomarkers for this autoimmune and degenerative disease of the central nervous system [7].

## Microglia and neurological disease

Microglia, the resident immune cells of the central nervous system, play a crucial role in neuroinflammation in response to various stimuli such as infection, injury, or neurodegeneration. Understanding the intricate interplay between microglia and neuroinflammation is essential for developing effective therapeutic strategies to combat debilitating neurological diseases. Zhao et al summarized the cross talk between microglia and Müller glia in the context of age-related macular degeneration (AMD), a progressive neurodegenerative disease that leads to photoreceptor demise and vision impairments. By elucidating the mechanisms underlying their interaction, the paper offers insights into potential therapeutic strategies targeting this interplay to mitigate AMD progression, and enhanced our understanding of the complex cellular interactions underlying AMD pathogenesis [8]. In another study, Huang et al provided an overview of the glycometabolic reprogramming of microglia in neurodegenerative diseases (ND), such as Alzheimer's disease (AD), Parkinson's disease (PD), and Amyotrophic lateral sclerosis (ALS). This review contributes to our understanding of the complex crosstalk between neuroinflammation, microglial function, and glucose metabolism in ND. By exploring the molecular mechanisms underlying glycometabolic reprogramming in microglia, the paper proposed potential therapeutic targets for the development of novel treatments for ND [9].

Tuberculous meningitis (TBM) is the most severe complication of tuberculosis (TB) with high rates of disability and mortality. Microglia plays a pivotal role in combating pathogens and maintaining brain homeostasis through various functions. Lu et al provided valuable insights into the relationship between microglia and TBM pathogenesis. By elucidating the roles of microglia in TBM and discussing potential HDT approaches, the review offers a comprehensive overview of therapeutic strategies for managing this devastating condition [10]. Recently, the voltage-gated proton channel Hv1 has been identified as selectively expressed in microglia in the CNS and played multifaceted roles in maintaining microglial function via regulating intracellular pH and production of reactive oxygen species. Starting with an examination of the molecular mechanisms underlying demyelination, Tang et al specifically focuses on the role of Hv1 in myelin repair and its therapeutic potential in CNS demyelinating diseases. By elucidating the involvement of Hv1 in microglial function and myelin repair processes, the paper offers insights into potential therapeutic strategies for addressing demyelinating disorders [11]. Sun et al reviewed relevant evidence from the literature on the crosstalk between microglia and astrocytes in migraine. By summarizing the potential crosstalk pathways between these two cell types, the review provides novel insights and ideas for future research and treatment approaches for migraine, contributing to our understanding of the complex interactions between microglia and astrocytes in migraine pathophysiology [12].

Both clinical and animal studies linked microglia with general anesthesia, understanding the mechanisms by which general anesthetic agents (GAAs) regulate microglial function is essential for mitigating the incidence of postoperative adverse effects. Yang et al provides a comprehensive overview of the actions of GAAs on microglia and the resulting changes in microglial function, shedding light on how GAAs interact with neurons via microglia to modulate nervous system function. This review enhances our understanding of the complex interplay between GAAs, microglia, and neuronal function, with potential implications for improving perioperative care and reducing the risk of postoperative cognitive dysfunction [13].

A study performed by Li et al investigated the role of TMEM166 in ischemic stroke following carotid endarterectomy (CEA) using a middle cerebral artery occlusion (MCAO) model. They found a positive correlation between microglial derived TMEM166 and the degree of ischemic brain injury. Transfection with Ad5-TMEM166 exacerbated ischemic brain injury by inducing microglial autophagy activation and release of inflammatory cytokines. Furthermore, TMEM166 deficiency was found to reduce brain inflammation and inhibit excessive microglial autophagy through the mammalian target of rapamycin (mTOR) pathway. These findings suggest that TMEM166 may play a crucial role in the development of ischemic injury after CEA and could serve as a biomarker for assessing the risk of postoperative ischemic stroke [14].

## Stem cell and extracellular vesicle-based therapies for stroke

Stem cells and extracellular vesicle-based therapies are widely studied for the treatment of various neurological disorders. These therapeutic approaches harness the regenerative potential of stem cells to repair damaged tissues and modulate inflammatory responses, while utilizing bioactive molecules released by extracellular vesicles to promote cellular regeneration and protect injured neurons. This minireview written by Xu et al introduces mesenchymal stem cell-based cell-free therapy as a promising therapeutic strategy, leveraging the immunomodulatory properties of mesenchymal stem cells to regulate microglia and astrocytes, modulate peripheral neuroninflammation, and deliver targeted anti-inflammatory interventions tailored to ND. The review also discusses engineering and safety considerations associated with this innovative therapeutic approach [15]. Osteoarthritis is a prevalent degenerative condition that affects a younger population and leads to high disability rates. An et al discusses the potential of mesenchymal stem cells (MSCs) and Radix Achyranthis Bidentatae (AB) in the treatment of Osteoarthritis by reviewing the roles of MSCs and AB in treating osteoarthritis, focusing on apoptosis reduction, chondrocyte growth, bone enhancement, angiogenesis, and regulation of estrogen and intestinal flora. It also explores the potential synergistic relationship between MSCs and AB, suggesting that AB may stimulate MSC repair for treating osteoarthritis [16].

Recent studies have demonstrated that the therapeutic potential of astrocytes-derived extracellular vesicles (ADEVs) for stroke. Wang et al provided an overview of the effects and functions of ADEVs on stroke recovery, discusses the prospects for utilizing ADEVs as a novel therapeutic approach for stroke treatment. By elucidating the role of ADEVs in stroke pathophysiology and recovery, the review offers insights into the mechanisms underlying their beneficial effects [17]. Using a mouse model of MCAO, Pan and colleagues administered PKH26-labeled M2 microglial EVs intravenously and evaluated their effects on stroke outcomes. Their results demonstrated that M2 microglial EV treatment reduced brain infarction and edema volume, improved neurological function, and decreased IgG leakage post-stroke. Further experiments showed that miR-23a-5p, delivered by M2 microglial EVs, targeted TNF and regulated MMP3 and NFκB p65 expression. This resulted in increasing BBB integrity. These findings provide valuable insights into potential therapeutic strategies for preserving BBB integrity and improving stroke outcomes [18].

## Inflammation and ischemic diseases

Inflammation plays a pivotal role in the pathogenesis of various diseases. The body's inflammatory response, while designed to protect against harmful stimuli, can become dysregulated, leading to tissue damage and exacerbation of disease progression. Targeting inflammatory pathways holds promise for attenuating disease severity and improving patient outcomes. The review written by Huang et al discusses intervention strategies targeting pannexin1 channels as a promising approach to alleviate inflammation after acute ischemic stroke, potentially improving clinical outcomes for affected patients. Additionally, the review proposes the use of brain organoid-on-a-chip technology to screen for microRNAs that exclusively target the pannexin1 channel, providing new therapeutic avenues for targeted regulation of pannexin1-mediated inflammation in ischemic stroke [19]. Chen et al investigated the role of thioredoxin1 (Txn1), a distinct RNA-binding protein (RBP) identified after intracerebral hemorrhage (ICH), in secondary injury and outcome improvement. The researchers observed that Txn1 was primarily expressed in microglia and neurons in the central nervous system, with reduced expression in perihematomal tissue after ICH. Overexpression of Txn1 was found to reduce secondary injury and improve outcomes in the ICH rat model by binding to metastasis-associated lung adenocarcinoma transcript 1 (MALAT1), resulting in reduced inflammation and apoptosis. This study suggests that Txn1 may serve as a potential therapeutic target for alleviating ICH-induced brain injury by modulating inflammation and apoptosis pathways [20]. Ducza et al consolidated existing literature on the role of NLRP2 inflammasome in the nervous system. They provided an algorithm-based protein network of human NLRP2, which could elucidate valuable molecular partnerships and pave the way for future therapeutic considerations. This paper contributes to advancing our understanding of neuroinflammation by shedding light on the less explored NLRP2 inflammasome [21]. For myocardial ischemia, the review written by Peng et al provides a comprehensive overview of the molecular mechanisms involved in myocardial ischemia injury, focusing on the intricate relationships between ROS, autophagy, and metabolism. By elucidating these pathways, it provides valuable insights into potential therapeutic targets for mitigating myocardial ischemia and improving patient outcomes [22].

Both genetic and environmental factors are known to influence Parkinson’s Disease pathogenesis. Jin et al focuses on the constitution of presynaptic vesicles related to DA homeostasis and discusses genetic and environmental evidence implicating presynaptic dysfunction in PD risk. It summarizes alterations in synaptic vesicular proteins that may contribute to the vulnerability of certain DA neurons to neurodegenerative changes. The review underscores the need for further research into the recently discovered mechanisms of vesicular dynamics in PD, which may lead to a deeper understanding of the disease and the development of novel therapeutic interventions for PD patients [23].

Su et al. highlights the emerging understanding of lipid-accumulated reactive astrocytes (LARAs) as a critical cell type in temporal lobe epilepsy (TLE) lesions. LARAs not only contribute to abnormal lipid accumulation within epileptic foci but also reduce the seizure threshold through upregulated activation of the adenosine A2A receptor (A2AR). Additionally, disruptions in mitochondrial oxidative phosphorylation (OxPhos) have been identified as significant contributors to lipid accumulation in astrocytes. Overall, this passage underscores the complex interplay between astrocyte dysfunction, mitochondrial impairment, and neuroinflammation in TLE, highlighting potential avenues for further research and therapeutic intervention in this debilitating neurological disorder [24].

## Blood flow and diseases

Blood circulation is crucial for delivering oxygen, nutrients, and immune cells to tissues throughout the body. However, disruptions in circulatory flow can have profound implications for health, contributing to the pathogenesis of various diseases. Conditions such as atherosclerosis, hypertension, and thrombosis can impair blood flow, leading to tissue ischemia, organ dysfunction, and increased risk of cardiovascular events. Using a rat model of chronic cerebral hypoperfusion-induced Vascular Cognitive Impairment (VCI) via two-vessel occlusion (2VO), Lin et al observed alterations in the number of pericytes in the brain following 2VO, indicating pericyte dysfunction. Additionally, they noted increased capillary constrictions at pericyte bodies in the brains of 2VO-induced rats, along with reduced cerebral blood flow (CBF). Increasing capillary lumens by CGS21680, a specific adenosine A2A subtype receptor agonist, led to substantial improvements in white matter repair and cognitive recovery. The study enhanced our understanding of VCI pathogenesis and offers prospects for targeted interventions in VCI [25].

Gagliano et al evaluated the impact of individual risk factors on vascular health by assessing pulse wave velocity (PWV) and augmentation index (AI) in a cohort of adults without modifiable cardiovascular risk factors and analyzing their role in accelerating vascular aging. The researchers conducted a secondary analysis of a Swiss population-based research project conducted between 2017 and 2018, involving 1097 participants. They found that individuals with hypertension, diabetes, and obesity exhibited higher PWV values, providing reference values for PWV and AI in individuals without modifiable cardiovascular risk factors and offers nomograms for risk score stratification, allowing assessment of the impact of individual risk factors on vascular health. These findings contribute to a better understanding of vascular aging and may aid in risk assessment and management strategies for cardiovascular disease [26].

Zhao et al underscores the evolving understanding of venous reflux in critically ill patients and emphasizes the clinical significance of assessing venous reflux status, particularly in the context of maintaining adequate cardiac output and mean arterial pressure. The authors proposed a comprehensive bedside ultrasonographic evaluation method that spans various venous territories, starting from the hepatic veins and progressing through the portal, renal and intrarenal, femoral, and pulmonary veins. Overall, this review underscores the importance of venous reflux status assessment as a complementary tool to traditional hemodynamic monitoring in the management of critically ill patients. In addition, The proposed bedside ultrasonographic evaluation method offers a practical approach for clinicians to assess venous reflux status and optimize patient care in the critical care setting [27].
